# What systemic factors contribute to collaboration between primary care and public health sectors? An interpretive descriptive study

**DOI:** 10.1186/s12913-017-2730-1

**Published:** 2017-12-01

**Authors:** Sabrina T. Wong, Marjorie MacDonald, Ruth Martin-Misener, Donna Meagher-Stewart, Linda O’Mara, Ruta K. Valaitis

**Affiliations:** 1 School of Nursing, University of British Columbia, 2211 Wesbrook Mall, T161, Vancouver, Canada; 2 Centre for Health Services and Policy Research, University of British Columbia, 201-2206 East Mall, Vancouver, Canada; 3 School of Nursing, University of Victoria, HSD B220, 3800 Finnerty Road, Victoria, BC V8P 5C2 Canada; 4 Dalhousie University, Room G26, Forrest Bldg., 5869 University Avenue, PO Box 15000, Halifax, NS B3H 4R2 Canada; 5 School of Nursing Health Sciences Center Room 3N25E, McMaster University, 1280 Main Street West, Hamilton, ON L8S 4K1 Canada

**Keywords:** Primary health care, Canada, Health system, Health services delivery, Qualitative

## Abstract

**Background:**

Purposefully building stronger collaborations between primary care (PC) and public health (PH) is one approach to strengthening primary health care. The purpose of this paper is to report: 1) what systemic factors influence collaborations between PC and PH; and 2) how systemic factors interact and could influence collaboration.

**Methods:**

This interpretive descriptive study used purposive and snowball sampling to recruit and conduct interviews with PC and PH key informants in British Columbia (*n* = 20), Ontario (*n* = 19), and Nova Scotia (*n* = 21), Canada. Other participants (*n* = 14) were knowledgeable about collaborations and were located in various Canadian provinces or working at a national level. Data were organized into codes and thematic analysis was completed using NVivo. The frequency of “sources” (individual transcripts), “references” (quotes), and matrix queries were used to identify potential relationships between factors.

**Results:**

We conducted a total of 70 in-depth interviews with 74 participants working in either PC (*n* = 33) or PH (*n* = 32), both PC and PH (*n* = 7), or neither sector (*n* = 2). Participant roles included direct service providers (*n* = 17), senior program managers (*n* = 14), executive officers (*n* = 11), and middle managers (*n* = 10). Seven systemic factors for collaboration were identified: 1) health service structures that promote collaboration; 2) funding models and financial incentives supporting collaboration; 3) governmental and regulatory policies and mandates for collaboration; 4) power relations; 5) harmonized information and communication infrastructure; 6) targeted professional education; and 7) formal systems leaders as collaborative champions.

**Conclusions:**

Most themes were discussed with equal frequency between PC and PH. An assessment of the system level context (i.e., provincial and regional organization and funding of PC and PH, history of government in successful implementation of health care reform, etc) along with these seven system level factors could assist other jurisdictions in moving towards increased PC and PH collaboration. There was some variation in the importance of the themes across provinces. British Columbia participants more frequently discussed system structures that could promote collaboration, power relations, harmonized information and communication structures, formal systems leaders as collaboration champions and targeted professional education. Ontario participants most frequently discussed governmental and regulatory policies and mandates for collaboration.

## Background

Strengthening primary health care is an important foundation towards building a more equitable and accessible system of care with better population health outcomes at reduced cost [1–4]. One approach to strengthening primary health care is to purposefully build stronger collaborations between primary care (PC) and public health (PH) sectors [5]. Over two decades of mainly descriptive research at local levels suggests there are numerous factors at multiple levels that can determine whether collaboration between PC and PH is successful [1, 6, 7]. Martin-Misener and colleagues’ [1] scoping literature review of collaboration between PC and PH found that successful collaboration between these two health sectors was related to systemic, organizational, and interpersonal factors. Collaboration at the systemic level is defined as the provincial and national level environment beyond the organization [8] and is related to improved health-related outcomes, reduced health disparities and increased access to health services [6]. At the organizational level, collaboration is defined as health professionals’ positive feelings of being part of the team [7], co-location of the team [9], implementation of new collaborative initiatives [10] and sustainable programs [6]. Finally, at the interpersonal level, collaboration between health professionals is related to improved patient health-related behaviors and the team’s increased capacity and expertise [6].

The few studies that have examined PC and PH collaboration has taken place at the individual (inter- or intrapersonal) level [11]. More work is required at the systemic and organizational levels to understand the contribution of these factors to collaborations generally [9], or between PC and PH [1, 12]. Indeed, a scoping review on collaboration between PC and PH [1] suggested that systemic factors, such as policy supports and resources, are needed to facilitate the development and sustainability of collaboration if the impact of collaboration is to extend beyond individual treatment to population health improvement. Provincial or national level polices that support collaboration can create social, cultural, and educational environments that could reduce the silos between PC and PH. For example, policies supporting inter-professional team-based care can begin to address the nature of “siloed” care, providing the impetus to address power differences that may exist between health professionals [8]. Acknowledgement by these system level policies for health professionals’ autonomy, working to their full scope of practice, and their ability to strengthen collaboration help set a stage for increased collaboration across sectors. Moreover, policies that shift how we educate health professionals for collaborative practice can help students recognize the values and responsibilities of their respective professions while instructing them in professional plurality [9, 13]. The purpose of this paper is to report the perspectives from key stakeholder interviews on: 1) what systemic factors, provincial or national, influence collaborations between PC and PH; and 2) how systemic factors interact to influence collaboration across these two health sectors.

## Methods

This was an interpretive descriptive [14] study design. The data collected and reported here were part of a four year, multi-province (British Columbia-BC, Ontario-ON, and Nova Scotia-NS, Canada) program of research examining structures and processes required to build successful collaborations between PH and PC at the systemic, organizational, and inter-, intrapersonal levels. In-depth interviews with key informants were conducted across the three provinces and at the national level.

Within Canada, provinces are responsible for organizing, delivering, and financing health care. There is also some federal transfer of funds to provinces for health care delivery. Each province has different divisions or departments of health; In BC, ON, and NS, PC and PH service delivery fall under different departments. A key difference between these provinces is the variety of PC delivery models that exist in ON compared to BC and NS [11, 12, 15]. Primary care in BC is mostly provided by fee-for-service physicians in solo and group practices who are geographically linked to a division of family practice. Less commonly, health authorities also deliver PC through community health centres and specialized clinics (i.e., youth health, STI/HIV diagnosis and treatment) often by nurse practitioners. In ON, there are 11 models of primary care delivery, such as solo and group fee-for-service physician practices and salaried community health centres, nurse practitioner-led clinics, and family health teams. Fee-for-service solo and group practice physician models are common in NS but there are a growing number of interdisciplinary teams, particularly in rural areas. In public health, provincial funding flows to regional jurisdictions to deliver services. British Columbia and NS have regional health authorities that provide public health programs and services whereas ON has public health units.

### Participants

Stratified purposive sampling [16] was used to recruit participants who represented policy-makers, managers, and inter-professional providers including nurse practitioners, public health nurses, registered nurses, primary care physicians, nutritionists. Participants were also identified and recruited using the snowball technique [16]. Eligibility criteria included: providing direct care or having responsibility for how services in PC or PH are organized or delivered, or having knowledge of collaboration between these sectors. Participants at the national level were representatives of organizations with a national presence or key informants representing agencies that were outside of the three study provinces.

### Recruitment and interviews

Potential participants were emailed inviting them to reflect on their knowledge and experiences regarding building and maintaining collaborations between PC and PH as a means to strengthen primary health care. Upon obtaining informed consent, participants answered a short demographic questionnaire about how long they had been in their current positions, age, and gender. This was followed by interviews, which lasted between 45 and 90 min, conducted by research team members, either face-to-face or over the telephone. All participants were given the interview guide and a sheet containing information on how we defined PC, PH, and collaborations (see Table 1) ahead of the interviews. Prompts were used where needed to explore the systemic factors; for example: “At a systems level, what fosters/limits building and/or maintaining such collaborations (Prompts: social, economic, political, health environments, policies; funding structures; legislation)?” All interviews were taped and transcribed verbatim. All procedures were approved by the researchers’ respective University Institutional Review boards and relevant health authorities who have their own ethics review boards for studies taking place within their jurisdiction.

**Table 1:** Definitions

*Primary care*: “…the crucial foundation of a health care system; Key features of primary care include the first point of entry to a health care system, the provider of person-focused care (not disease oriented) over time for all but the most uncommon conditions and the part of the system that integrates or co-ordinates care provided elsewhere or by others.” [2]
*Public health*: “…an organized activity of society to promote, protect and improve, and when necessary, restore the health of individuals, specified groups, or the entire population. It is a combination of sciences, skills, and values that function through collective societal activities and involve programs, services, and institutions aimed at protecting and improving the health of all people. The term “public health” can describe a concept, a social institution, a set of scientific and professional disciplines and technologies, and a form of practice. It is a way of thinking, a set of disciplines, an institution of society, and a manner of practice. It has increasing number and variety of specializes domains and demands of its practitioners [and] increasing array of skills and expertise.” [32]
*Collaboration*: “a recognized relationship among different sectors or groups, which have been formed to take action on an issue in a way that is more effective or sustainable than might be achieved by [any one group or sector] acting alone.” [32, 33]

### Analysis

We conducted an interpretive content analysis of the interview data using procedures for qualitatively derived data [17, 18]. Transcripts were repeatedly read by the members of the investigative team to identify patterns in the data. NVivo [19] was used to organize and support coding and analysis of the data. All authors contributed to coding which started with holistic coding; As more data were collected and analyzed, coding categories were refined. Next, provincial teams independently coded assigned transcripts before meeting with the full team. Following the practice of using conventional grounded theory analytic techniques as pragmatic ‘tools’ in interpretive qualitative inquiry, the process of constant comparison [20] guided the analyses of this study since the aim was to explore and uncover commonalities and patterns, and to understand social phenomena [16, 18, 20]. After this first-level coding was conducted, the team categorized these codes into second-level codes or pattern codes and generated a draft code book [21]. All transcripts were coded by at least two members of the research team.

We examined the frequency of “sources” (individual transcripts) as well as references (coded text excerpts) within sources to assist in deriving the factors. Matrix queries were conducted in NVivo to determine any potential differences by sector and province and identify potential relationships between codes at the systems level. “NEAR content” matrix queries were used to identify text passages in which text was coded as one factor and located ‘near’ text coded for another factor. Data from these searches were then examined to explore potential relationships among the codes. A manual search of these text passages was done to find potential quotes that showed the presence of relationships between factors.

## Results

We conducted a total of 70 in-depth interviews with 74 participants who were working in either PC (*n* = 33), PH (*n* = 32), both sectors (*n* = 7), or neither sector (*n* = 2). Those who reported not working in either sector were health services researchers or educators. Participant roles were diverse, and included direct service providers (*n* = 17), senior program managers (*n* = 14), executive officers (*n* = 11), and middle managers (*n* = 10). The majority of participants were women (*n* = 58) with similar numbers of interviews being completed across BC (*n* = 20), ON (*n* = 19), and NS (*n* = 21). The remaining participants (*n* = 14) were working in or knowledgeable about collaborations in PH and PC but were located in other Canadian provinces, or working at a national level.

Our analysis suggests there were a total of seven systemic factors that influence collaboration: 1) health service structures; 2) funding models and financial incentives; 3) governmental and regulatory policies and mandates; 4) power relations; 5) harmonized information and communication infrastructure; 6) targeted professional education; and 7) formal systems leaders as collaborative champions. Table 2 lists the influencing factors, from most to least commonly occurring, and the differences found among provinces, and between PC and PH sectors, are highlighted and discussed further below.

**Table 2:** Systemic factors influencing public health and primary care collaboration

Factor [add row] Health service delivery structures	Element [add row] Infrastructure to support collaboration (e.g. Information Technology, physical space); Opportunities for PC and PH to transcend silos (e.g. inter-branch/divisions/department committees); Shared PC PH portfolios
Funding models and financial incentives	Increased/sufficient allocation of financial resources for collaboration; Alignment of funding models and incentives for public health and primary care collaboration; Potential strategies of funding collaboration (e.g., secondments, incentives, fee codes)
Governmental and regulatory policies and mandates for collaboration	Expectations that partners are essential; Clear governmental policies, mandates for collaboration; Consistency of standards around collaboration for public health and primary care; Expectations/accountability for reporting on collaborations using common quality indicators
Power Relations	Leveling the playing field; Turf protection
Harmonized Information and Communication Infrastructure	Clear and effective information and communication infrastructures; Interoperable public health and primary care communication systems and electronic record systems (Electronic Medical Record; Electronic Health Records)
Formal Systems Leaders as Collaborative Champions	Identification and formalization of systems leaders; Leadership for collaboration; Long-term strategy for collaboration; Leadership understanding of benefits of collaboration.
Targeted Professional Education:	Educating new professionals for collaboration between public health and primary care; Continuous professional development for collaboration between public health and primary care

### Health services delivery structures that promote PC and PH collaboration

Both PC and PH participants discussed the importance of health services structures in promoting collaboration. However, queries by province suggest that BC participants perceived this factor as more important than either ON or NS participants. Three elements in this factor include: *infrastructure to support collaboration*, *structures and mechanisms for public health and primary care to transcend silos*, and shared PC PH portfolios. All elements were expressed as barriers to collaboration. Ministries of Health purposely created structures that provided opportunities for collaboration. For example, mandatory reporting of sexually transmitted infections that requires partner notification, and the provision of immunizations, are areas where structures already exist in the three provinces. What promotes collaboration between these two sectors are mechanisms for who (e.g., PH nurse or PC office) will complete the partner notification or explore benefits of collaboration in the case of immunizations.

Participants noted that having a clear governance structure and mechanisms that align with provincial directions on collaboration can facilitate stronger links between the PC and PH sectors. This participant described important structural configurations, two departments in the provincial government, that reinforce silos and may prevent collaboration,

“*I am quite closely associated with PC, with their policy advisor, and also traditionally probably wasn’t as closely aligned with PH in just the way the organization works within government… we have the 2 departments which has been a little bit of a challenge. The Department of Health which looks after PC, preventive care. A lot of things that we would call within PH. You know, illness prevention and PC, primary healthcare prevention, that sort of thing. Public safety and infection prevention, disease prevention, that focuses a little bit more on the various determinants of health. Some of that sits over at Health Promotion and Protection. As we are fond of saying, 2 departments, 1 system.*” [NS 16]

Moreover, participants suggested that provincially mandated governance structures at regional or local levels explicating a team-based approach, or policies to provide direction on implementing models of care that support interprofessional collaboration, would be helpful. While these kinds of local structures exist in ON (e.g. Family Health Teams), they remain relatively scant in BC and NS.

### Funding models and financial incentives supporting collaboration

Many participants discussed the important role of funding and financing in facilitating collaboration between PC and PH. Both BC and ON participants discussed this factor more than NS interviewees and PC participants discussed funding and incentives more than their PH counterparts. We categorized three elements for this factor: *increased/sufficient allocation of financial resources for collaboration; alignment of funding models and incentives for PC and PH collaboration;* and *potential strategies of funding collaboration.*

Participants recognized that additional financial resources could strengthen collaboration between PC and PH. Provincial funding used for paying health professionals to work together in the health care system was one suggestion to support collaboration. Participants also discussed how funding new staff positions, where part of the role would be to increase collaboration purposefully, could create systemic changes in the delivery of health services. As one participant stated,

“*it [collaboration] happens because someone is willing to pay for it and willing to support the infrastructure to make it happen…you are not going to get pharmacists in physician offices unless they are paid*” [NS 13]

Another example is the willingness of provinces to pay for and position nurses within PC practices to increase collaboration with PH, as well as deliver PH services.

Participants discussed the importance of funding being aligned with provincial, and less often federal, policies. Provincial policies, such as the implementation of nurse practitioners to deliver PC using a population health approach, create the impetus to flow funding to regional jurisdictions. This participant speaks of funding becoming available to specific health units,

“*….the nurse practitioners were brought in specifically…..because that funding became available to the [public] health unit.… for the pre and postnatal project that had the nine sites......there was a pretty strong link to the Healthy Babies, Healthy Children program…..the [provincial] Ministry obviously supported with funding*.” [ON 15]

This quote also suggests a potential strategy for funding collaboration at the systemic level was for provincial ministries of health to work with regional jurisdictions or negotiate with the Federal government (e.g. a federal Primary Health Care Transition Fund) to allocate funding to support policy related to increasing collaboration between PC and PH.

Participants recognized that current funding structures, such as fee-for-service (FFS) and salaries for physicians, were well established across Canada. However, they suggested funding strategies such as the introduction of incentive billing codes designated for inter-professional collaboration could enable stronger and more frequent collaboration. Another suggestion from PC participants was to provide some combination of salary and FFS billing to facilitate collaboration. As this participant points out, providers need some incentive to work together,

“*the way people are reimbursed is going to be a problem as far as doctors go in some places…PC physicians…they aren't going to want to take time out of their day from seeing patients to put an effort into PC prevention and collaboration if it is going to take away from their billing*.” [NS 19]

The above quote suggests incremental changes (e.g., incentive billing codes) implemented at the provincial level may be able to persuade increased collaborative behavior of physicians at the intrapersonal level. Moreover, in attempts to strengthen collaboration, it was incumbent upon the insurer (e.g., the province) to make the business case that FFS health care providers would not be financially disadvantaged if they were to collaborate with non-FFS counterparts. Put another way, FFS encourages individual billing and “efficient” (e.g. visits shorter than 15 min for each patient) throughputs versus collaboration to address a patient’s health and healthcare needs.

### Governmental & regulatory policies & mandates for collaboration

Ontario participants identified the importance of policies and mandates at the provincial level more often than those in BC or NS. Public health participants discussed this factor more frequently than their PC counterparts. There were four interconnected elements of this influencing factor: *expectations that partners are essential; clear governmental policies, mandates for collaboration; consistency of standards around collaboration for PC and PH;* and *expectations/accountability for reporting on collaborations using common quality indicators.*

Participants identified an increased need for government policies mandating collaboration between PC and PH. Leadership at the provincial level is necessary to deliver a vision of collaboration and partnerships for jurisdictional (e.g., health authorities) policies and mandates to be influenced. As one participant notes, provincial governments can support collaboration by providing direction on how they would see organizations working together,

*“…provincial policy…different branches with that ministry have to really believe in this and support it. Write policy that says these organizations are going to work together….really important that the provincial policy be supportive...allowing it to happen rather than putting up road blocks.”* [ON 02]

In addition, participants identified that the divisions within the provincial ministries responsible for PC and PH service delivery need to develop consistent standards to strengthen collaboration. Participants provided examples of PH nurses and nurse practitioners caring for patients who had a regular PC provider but were unable to access needed health services or were being seen by PH for another issue (e.g., immunization). Whereas the PH standard was to communicate with PC, no such standard to exchange information existed within PC.

Participants also suggested the government could understand whether collaboration was occurring at the system level by measuring and reporting the degree to which PC and PH were improving health system and patient reported outcomes together,

*“…it is possible the linked database allows us…a fair amount of opportunity to measure and…..[monitor collaboration], we don't use that enough…there are greater opportunities for evaluating the effectiveness of the current system……of bringing people together in a new way.”* [BC 03].

In making system changes for collaboration, participants expected there be evaluation of whether these changes were beneficial.

### Power relations

Structures at the systemic level, meant to improve the health of the population, decrease or increase power struggles between the two sectors. Participants in BC discussed systemic power relations more often than either ON or NS interviewees, and discussed this factor similarly between PC and PH. Power relations between PC and PH act as a strong force to keep the status quo, thus presenting barriers to collaboration. Two elements were evident: *leveling the playing field* and *turf protection*.

The disparity in resources between PC and PH perpetuates service delivery and workforce silos. Provincial ministries of health have tried to address unequal distribution of power (resources and funding) by creating positions in which the portfolio includes responsibilities and oversight of service delivery or program implementation in both PC and PH. The following quote illustrates the day-to-day differences between these health sectors with respect to health human resources and funding,

*“PH and PC don't really have a level playing field in terms of funding, public image and…attention…for any kind of collaboration it's nice for there to be equal power…the PC system has a lot more money and a lot more people than the PH system does”* [ON 13].

Both the PC and PH sectors enact power to protect what they see as their turf. In the case of delivering flu vaccine or immunizations, both sectors feel responsible for delivering these services,

*“It’s [using PH to deliver immunizations] usually cheaper, it’s a better way to go but, you know, a lot of family docs don’t want to give up on what they see as a fundamental part of PC….so you’ve got a combination of turf wars and funding wars and sort of reporting record keeping, administrative wars*.” [BC 06].

This quote illustrates the need for PC to protect its ability to deliver this kind of service since it is tied to an ‘easy’ source of funding (versus billing for working with patients who have multiple chronic conditions) and meets their goal for delivering health prevention. In this case of a dual provider system, that both PH and PC deliver immunizations, power is enacted through turf protection, separate information systems and funding split between PH and billings in PC. Systemic challenges to collaboration are exacerbated by factors such as lack of shared space and irregular communication.

### Harmonized information & communication infrastructure

The systemic influence of having an information infrastructure and mandates for using different modes of communication was important. Having a harmonized information infrastructure was most frequently discussed by BC and PC participants. There were two elements in this theme: *clear and effective information and communication infrastructures* and *interoperable PC and PH electronic record systems*.

Each sector’s ability to use existing communication and information infrastructures influenced collaboration. Participants used technology (e.g., email, fax) and structured communication forms such as SBAR technique (Situation, Background, Assessment Recommendation), to try and increase the exchange of clear and effective information. Systemic investment in information technology systems was required to ensure that the modes of communication for sharing patient information were secure and the privacy of the patient was maintained.

Participants recognized that the lack of information sharing between the two sectors could negatively influence collaboration. As one participant pointed out:

“*[if] physicians would have access in some way to Panorama [public health electronic information system] they could then put their information into it…they have a record of the immunization that they could access easily at any time.”* [NS 02].

Interoperability of information systems and use of provincial or pan-Canadian systems was viewed as an opportunity that could strengthen collaboration. However, many participants thought that since health is governed provincially, information interoperability was a particularly difficult challenge that would need to be addressed at the systemic level within and across jurisdictions.

### Formal systems leaders as collaborative champions

Having leadership at the provincial (system) level was important in promoting collaboration. This theme was discussed similarly amongst ON and NS participants but more frequently by participants in BC. PH participants discussed the need for formal system leaders more frequently than PC participants. Four elements captured the dialogue of participants including: *identification and formalization of systems leaders; leadership for collaboration; long-term strategy for collaboration;* and *leadership understanding of benefits of collaboration.* Formalizing system leaders through an appropriate governance structure at the provincial level to support collaboration between PC and PH was important. This quote captures participants’ perceptions on an environment unable to nurture collaboration,

*“it requires a great deal of wisdom at the level of the minister to make sure that people are assured. And I … I just don’t think that our political system enables that to happen at the moment.” [ON 10 PC]*

Participants noted the lack of a long-term strategy, either at the provincial or federal level, for strengthening collaboration across the two health sectors. Participants felt that some leaders were unaware of the tangible benefits of systemic collaboration,

*“You need to have policy change in order to have system change. My concern is that the people who are writing the policy aren’t understanding what the real issues are, and they are not making policy that is actually going to change things for people’s lives” [NS 05].*

Moreover, participants were concerned that system level policies or mandates needed direct input from leaders within both PC and PH in order to be effective policy directors.

### Targeted professional education

Participants believed that the system could strengthen collaboration by providing guidance and program levers to influence inter-professional education. This theme was, again, more frequently discussed in BC than the other provinces, although both PC and PH discussed the need for professional education with similar frequency. There were two elements for this influencing factor: *educating new professionals for collaboration between PC and PH;* and *continuous professional development for collaboration.*

Many participants believed both initial and ongoing training of health care providers could begin by breaking down how PC and PH have traditionally operated,

“*Have a good look at our core education and the education system in terms of building that capacity and valuing and understanding…You shouldn't come into healthcare if you are not prepared to be in a team…critical piece - I think the ongoing education…peer review is really important in terms of team work*.” [NS 18]

Participants believed that providers needed training on how to work together, in addition to their disciplinary and sector-specific knowledge, and named several areas that could be a common base of knowledge, such as how to collaborate, making good use of technologies (e.g., teleconferencing), respectful communication, population health concepts, social determinants of health, and team-based care. Participants also suggested more integrated academic and real world practice training that included both PC and PH would help facilitate collaboration; many suggested that learning about health professional’s scopes of practice issues also needed to be incorporated into provider’s training,

“*While we are transitioning to a whole new system because of shortages with physicians and nurses and all the other health system workers we need to look at a different way of working…what role can the physicians play in supporting and promoting that different way of practice*” [NS 15].

### Relationships between systemic influencing factors

We found relationships between influencing factors at the systemic level which speaks to the complexity of this field of study. Our analysis identified relationships between several systemic level factors, including: *governmental and regulatory policies and mandates* and *health care delivery structures* that promote collaboration and formal system leaders as collaborative champions; *health care delivery structures* and *funding models* and *harmonized information systems*; and *information systems* and *targeted professional education*.

We present here two quotes of relationships that were most apparent. This first quote illustrates the relationship between a province-wide mandate and models of service delivery structures with accountability for PC and PH collaboration. This participant suggests a province-wide mandate could be more than an invitation to participate but an expectation of transforming the PC and PH delivery systems to be more integrated,

*“… I think until we have a complete mandate ……for health services to be delivered through family health teams* [in Ontario]…..[only] *a hundred and fifty [*family health teams exist*] is nothing when you compare to like… Brazil with nearly thirty thousand of them…[*primary health care*] is expected to be delivered, not simply we invite you to participate by applying for a grant or whatever…it’s actually making it an expectation… that if you are going to be delivering primary health, then you do it through a family health team, which is collaborative internally first, not led by, you know, a particular quarterback. It’s really a team. And also it’s required there be… accountability to ensure that there’s linkages with PH.” (ON PH)*

As this participant suggests, different models of service delivery that encourage PC and PH collaboration are possible but require governmental policies and mandates. Formal leaders championing these service delivery structures, such as family health teams, can serve as a foundation for expectations about what services are delivered, who delivers the services, and how team members with complimentary expertise work together.

Not surprisingly, health service delivery structures that promote collaboration between PC and PH need funding. Without these systemic influences, collaboration occurs sporadically at the interpersonal level until there is a provincial or pan-Canadian need to communicate,

*“… a good example of this was SARS…there was fairly public criticism about this, when you’re in the middle of an emergency suddenly you realize that there actually aren’t harmonized communication vehicles. So, for instance, PH needing to get out messages with regard to the implications for individual practitioners… And really there isn’t a harmonized communication system to allow for that.” (Nat PH)*

This quote also points to the need for PC and PH sectors to receive ongoing education on where to find information and how to collaborate in areas with common goals (i.e. communicable disease surveillance, prevention, and treatment).

## Discussion

Our analysis provides insight into seven common, yet distinct, systemic level influences across three Canadian provinces that are clearly interrelated. While all system level factors are important influences, some factors such as governmental mandates and health service delivery structures and funding, may be key to building stronger collaboration between PC and PH. The interrelationships between the systemic influences provides clues to what ingredients are needed at a systems level for successful PC and PH collaboration.

Similar to what is reported by Tomoaia-Cotisel et al. [22], the external environment or rather, the systemic context of each province, can account for the variation in emphasis of the importance of specific structures. Our results suggest there was some variation in the importance of the themes across provinces, where BC participants more frequently discussed system structures that could promote collaboration between PC and PH, power relations, harmonized information and communication structures, formal systems leaders as collaboration champions and targeted professional education. Ontario participants most frequently discussed governmental and regulatory policies and mandates for collaboration. Nova Scotia participants were most frequently in-between BC and ON.

Most themes were discussed with similar frequency between PC and PH, except where PH more frequently discussed the importance of formal systems leaders and the need for governmental mandates to facilitate collaboration between the two sectors. In part, it could be that PH participants were collectively voicing their concerns that health ministries place more emphasis on acute care system objectives than on population health objectives [23]. However, formal system leaders could provide opportunities to collaborate through joint work on policies or reforming the current delivery structures to take advantage of each sector’s expertise. Primary care and PH sectors, and those who educate these health professionals, could be held accountable through system levers such as funding and incentives that support educating about and delivery of services using collaboration.

Indeed, carrying on with “business as usual,” or rather, working in silos seemingly presents no immediate harms to patients or the population. Yet, the potential for reaching system goals of increased effectiveness, equity, and efficiency are possible by strengthening collaboration between PC and PH. At least 50 years of evidence suggests strengthening primary health care can result in improved health of the population and decreases in health system expenditures due to use of less expensive community-based care compared to acute care [3]. Collaboration between the sectors could be encouraged through governmental vision and policies calling for them to work together.

Undertaking reform to increase collaboration is complex. While we present power relations as a specific theme, it becomes operationalized across many different factors, as well as at the systemic, organizational, and individual level. At the systemic level, power relations between the sectors and the lack of disruption in systemic factors (e.g. teaching health professionals within their respective disciplines) acts as a strong lever to keep the status quo.

Team based models, supported by policy and teaching of health professionals, could be considered a disruption process to power relations. A recent review by Levesque and colleagues [12] suggests there are potential models of care such as Family Health Teams (Ontario, Canada) [24], Accountable Care Organizations (United States of America) [25], and Multidisciplinary Health Clinics (France) [12] that hold promise for strengthening collaboration between PC and PH. Notably, the implementation of models of care whose mission is to provide shared PC and PH activities were enabled by systemic factors such as funding, provincial and national mandates, and the establishment of formal system leaders. One example of a provincial mandate was found in Ontario where new legislation has been passed [26] to ‘put patients first.’ It argues for stronger systems integration and a focus on population health by building stronger links between PH and other health services.

Limitations of this study emerge from the recruitment process for participants. Since we focused on participants from three Canadian provinces, with the addition of a few informants from other provinces and at a national level, there may have been key informants from other provinces or territories with suggestions for other systemic factors needed for successful collaboration between PC and PH. Furthermore, the snowball sampling technique may have led to sampling of participants with similar views. However, the systemic influences identified in this study were supported by many respondents, and their experiences with these factors were not discussed with similar frequency, indicating that our sample was representative of people with varied experiences and views. The reliability of these results was enhanced by input from of our co-investigators and collaborators who were situated in each of the provinces and held positions within primary care or public health. Finally, these factors, except for health service delivery structures and formal systems leaders, were also reported in our scoping review [1].

## Conclusion

Our results suggest there are systemic factors associated with successful PC and PH collaboration. More could be done at the systemic level to strengthen collaboration between PC and PH: focusing on having formal system leaders who champion PC and PH collaboration, shifting funding to increase and strengthen collaboration between these two sectors and further implementation of interprofessional teams. An assessment of the system level context (i.e., provincial and regional organization and funding of PC and PH, history of government in successful implementation of health care reform, etc.) along with these seven system level influences could assist other jurisdictions in moving towards increased PC and PH collaboration.

When operating together, PC and PH sectors could potentially lead to transformative system learning and change. The “collaborative advantage” of PC and PH working together, where benefits accumulate from a group working together on a common goal [27], cannot materialize without systemic support in training future generations of clinicians. Notably, collaboration is considered to be an overarching, requisite process in effective clinical practice [28], in addition to successfully carrying out inter-professional education [29, 30]. Targeted education that includes attention to power dynamics, co-creation of actionable knowledge [31] and using each other’s existing knowledge [9] needs to be supported by additional infrastructure such as university curricular changes and health systems investing in collaborative learning communities of practice.

## Abbreviations

PC: Primary care
PH: Public health
